# The Hepatic Crown-like Structure: A Focal Point for Macrophage Evolution and Disease Response in Steatotic Liver Disease

**DOI:** 10.3390/livers6040072

**Published:** 2026-08-01

**Authors:** Kyle Yuquimpo, Ayobami Dare, Isabel Aranzazu Pulido Ruiz, Steven A. Weinman

**Affiliations:** Department of Internal Medicine and Liver Center, University of Kansas Medical Center, Kansas City, KS 66160, USA

**Keywords:** Kupffer cell, crown-like structure, hepatic crown-like structure, lipid-associated macrophage, steatotic liver disease

## Abstract

The hepatic crown-like structure (hCLS) is a shell-like aggregate of macrophages surrounding a large lipid-laden dying hepatocyte. This feature was initially assumed to simply be a response to increased inflammatory stress during steatotic liver disease, but recent studies have shown that the hCLS is a critical site for lipid processing, inflammation regulation, fibrosis modulation and macrophage development. Furthermore, advances in lineage tracing and transcriptomic analysis have provided information on the nature of the macrophage subtypes present in the hCLS. The hCLS consists of a heterogeneous mixture of macrophages that arise largely from bone marrow-derived infiltrating macrophages (IMs) but also have some of the properties of Kupffer cells (KCs). Most of the cells are variations of Lipid-Associated Macrophages (LAMs) expressing surface proteins such as GPNMB, TREM2, CD9, CD36, CD63 and CD11c. In addition, a class of LAM-like KCs is also present and these typically express many of the LAM proteins along with KC lineage proteins such as VSIG4 and CLEC4F. The hCLS plays an important role in lipid disposition and inflammation but conflicting evidence appears to support roles in fibrogenesis, extracellular matrix remodeling, and matrix degradation. This review aims to describe the critical findings and discoveries made regarding hCLSs and their role in macrophage development and function in steatotic liver diseases.

## 1. Introduction

The global incidence of chronic liver disease (CLD) has risen over the past decade, particularly in younger populations [1]. In the United States, CLD is one of the top ten leading causes of mortality [2,3]. Numerous etiologies can lead to CLD, and steatotic liver disease (SLD) makes up a large proportion. It is now recognized that SLD is a class of functionally related diseases (i.e., metabolic dysfunction- and alcohol-associated) that share similar histological characteristics and natural history [4,5]. Widespread micro- and macro-vesicular steatosis, pericellular fibrosis and hepatocyte ballooning are histological features unique to SLD progression and are not present in other forms of chronic liver disease such as those due to viral, autoimmune, or cholestatic processes. Over the past decade, the hepatic crown-like structure (hCLS), a collection of macrophages surrounding lipid-laden hepatocytes, has been identified as a feature uniquely present in SLD. Initially presumed to simply be a nonspecific response to cell damage, hCLSs in fact appear to be highly complex structures. Recent evidence regarding hCLSs has been conflicting, with some studies suggesting a pathogenic role [6–10] and others suggesting a protective role [11]. Recent advances in lineage tracing and single-cell sequencing have allowed for a clearer picture regarding the origin of the macrophages present in hCLSs. In this review, we will discuss macrophage diversity in SLD, the cellular composition and functions of the macrophages of the hCLS, and the role of hCLSs as unique sites of macrophage evolution in SLD.

## 2. Macrophage Ontogeny and Diversity in Steatotic Liver Disease

Prior to about 2010, all macrophages were presumed to arise from bone marrow hematopoiesis [12,13], but this notion was challenged by Schulz et al. [14] who showed that tissue-resident macrophages appear despite loss of MYB, a critical transcription factor for the development of bone marrow-derived macrophages. Similar ontological findings were further observed in human fetuses confirming the presence of macrophages with a separate ontological origin outside of the bone marrow [15]. These tissue-resident macrophages, particularly in liver, brain, epidermis, and lungs, were subsequently confirmed to arise from erythro-myeloid progenitors [16,17]. Based on these foundational studies, it is now accepted that hepatic resident macrophages called Kupffer cells (KCs) are ontologically distinct from other macrophages of the gastrointestinal system including those in the intestinal tissue. In the normal liver, these embryonically derived KCs (emKCs) persist throughout life and are maintained during homeostatic steady-state conditions without a need for replacement by bone marrow cells [18,19]. However, during liver disease or injury, monocyte-derived KCs (moKCs) originating from the bone marrow take residence in the liver [20–24]. Circulating blood monocytes are recruited into the liver during states of injury [25] by a process that involves signaling via the CCL2/CCR2 axis. The secretion of C-C motif ligand 2 (CCL2), also known as Monocyte Chemoattractant Protein 1, by injured hepatocytes and KCs [26] activates circulating monocytes by binding to CCR2. After tissue entry, these circulating monocytes become infiltrating macrophages (IMs) and can be subdivided based on LY6C, CX3CR1 and CCR2 expression in mice and CD14 and CD16 in humans [27]. IMs with both high CCR2 and LY6C protein levels exhibit a pro-inflammatory phenotype [28] while those with low CCR2, low LY6C, but high CX3CR1 represent less inflammatory patrolling monocytes that are present in the liver during steady-state conditions [29].

The transition of IMs toward an moKC phenotype under disease conditions has been elegantly described by several groups. Sakai and colleagues defined key epigenetic changes necessary to transform monocytes into moKCs [30] and Bonnardel et al. [31] showed the role of specific macrophage niches, defined by stellate cells, endothelial cells and hepatocytes, in allowing this differentiation to occur. The life span of these newly formed moKCs is still unclear, though some groups have suggested that once formed, they can persist and even self-renew [32,33].

Multiple studies have examined the phenotypic nature of macrophages in steatotic liver diseases and classifications of some of the disease-specific macrophages present have been proposed. Some authors have identified macrophages based on their associated disease process such as “Nonalcoholic Steatohepatitis-Associated Macrophages” (NAMs) [34], while others have referred to them by their surrounding histopathological findings such as “Lipid-Associated Macrophages” (LAMs) [35–38] or “Scar-Associated Macrophages” (SAMs) [39]. These nomenclature schemes may overlap and have led to some degree of confusion regarding the exact origin and function of different macrophage subsets in SLD. Moreover, the relationship between hCLSs and these disease-specific macrophage subsets is not well understood and warrants clarification. The characteristics of the different macrophage subsets observed in steatotic liver disease and as components of hCLSs are summarized in Tables 1 and 2.

## 3. Origin and Compositional Diversity of Hepatic Crown-like Structures

The term crown-like structure (CLS) refers to a specific structure of macrophages circumferentially encompassing a damaged or dying lipid-laden cell. The terminology was first used by Cinti et al. to describe this unique microscopic finding in the visceral fat of obese patients and in murine models of obesity [40]. hCLSs were later identified by Itoh et al. a decade later in a murine model of Metabolic-Dysfunction Associated Steatotic Liver Disease (MASLD). They were described as large, lipid-filled hepatocytes that, viewed in cross section, were surrounded by a ring of macrophages [6]. Since then, the terms hCLS and CLS have been used interchangeably to identify these uniquely positioned macrophage structures. The identity of these macrophages has been a substantial point of discussion which was initially limited by their identification via expression of pan-macrophage markers such as F4/80 and CD68, which made it impossible to delineate IMs from KCs, emKCs from moKCs, or the involvement of additional subgroups. New lineage tracing and transcriptomic technologies have now provided the ability to identify hepatic macrophages present in hCLSs based on their properties and ontological origin. In mice, the expression of TIM4, the product of the *Timd4* gene, appears to identify emKCs [20,33]. Conversely, CCR2, CX3CR1 and LY6C are restricted to bone marrow-derived monocytes and IMs [41]. Miura and colleagues showed that pharmacological inhibition of CCR2 in mice fed a steatohepatitis diet caused a loss of F4/80+ hCLSs [42], suggesting that CCR2 activation is necessary to form hCLSs. Recent evidence, however, has challenged this hypothesis. Daemen and colleagues showed that whole-body knockout of *Ccr2* in a murine metabolic dysfunction-associated steatohepatitis (MASH) model decreased the total number but did not eliminate hCLSs from the liver [11]. This suggests that CCR2 signaling may play a role in dictating the composition of hCLSs as opposed to being strictly required for their formation. In addition, many studies have concluded that hCLSs can be composed of macrophages expressing markers not typically associated with KCs including CD11c [6,7] and MINCLE/CLEC4E [43]. However, KC-specific proteins have also been observed in hCLS macrophages including SIGLEC-1 (also known as CD169) and CLEC4F [7], but studies using irradiated, bone marrow-transplanted mice show that even the KC-like CLS cells can be derived from bone marrow precursors [7]. Finally, CLSs have also been described in other tissues including epicardial adipose, breast adipose and omental tissue which are beyond the scope of this review [44–48].

### 3.1. Lipid-Associated Macrophages (LAMs)

LAMs are a subset of macrophages that have been described in multiple tissue sites with excessive lipid accumulation such as the vasculature, intestinal lamina propria, adipose tissue, and hepatic parenchyma [49]. These LAMs can be identified by their expression of specialized proteins including GPNMB, CD63, CD36, TREM2, SPP1, and CD9 [49]. They are generally thought to play a role in metabolizing lipids and suppressing tissue injury [38,49–51]. Daemen and colleagues observed that two classes of LAMs exist within hCLSs, one expressing high levels of CCR2 and CX3CR1 (c-LAMs) and another expressing low levels of CCR2 but high levels of CD63, CD9, TREM2, and GPNMB (conventional LAMs) [11]. They determined that c-LAMs are more monocyte-like and critical in macrophage aggregation. Moreover, it is likely that c-LAMs are more pro-inflammatory as in a liver MASH model, CCR2 knockdown prevented accumulation of inflammatory LY6C-high cells. It is unclear whether LAMs and c-LAMs originate from different precursors, but evidence has suggested that CCR2-low IMs/patrolling monocytes are in fact derivatives of CCR2-high IMs [52], suggesting that both types of LAMs could have a single origin. In the steatotic liver, LAMs expressing high levels of GPNMB and TREM2 have only been identified at hCLS sites and are not typically distributed as “normal” macrophages located within the hepatic sinusoid [49]. This suggests that expression of the LAM-specific proteins occurs due to signals specifically present at the site of hCLSs. Triggering Receptor Expressed on Myeloid Cells 2 (TREM2) warrants special consideration as it has not only been useful in identifying hCLSs but has been shown to play a functional role in LAMs [11,50,53,54]. Its knockdown in vitro has been shown to increase oxidative stress [55], suggesting that TREM2 may modulate macrophage responses to excessive lipids and reduce oxidative stress. In vivo, the loss of TREM2 in both whole-body and myeloid-specific knockout mice increased the accumulation of pro-inflammatory LY6C+/CX3CR1- IMs [54].

While TREM2-expressing macrophages appear to play a role in fibrosis, conflicting evidence exists on whether they are in fact driving fibrosis or preventing it. Ramachandran et al. [56] showed that, in human metabolic dysfunction-associated steatohepatitis (MASH)-related cirrhosis, CD9+/TREM2+ macrophages localized to areas of fibrosis. In co-cultures with stellate cells, these macrophages activated profibrotic responses [56]. Additionally, myeloid-specific Trem2 knockout in a MASH mouse model prevented the deposition of collagen [54] suggesting that TREM2+ macrophages may also play a role in collagen formation. Alternatively, other investigators have found that TREM2 is required in macrophages to prevent fibrosis progression in MASH models [50]. Overall, these data show that LAMs localize to the hCLS but are heterogeneous and appear to modulate both inflammation and fibrosis.

### 3.2. LAM-like Kupffer Cells (LLKCs)

Most evidence suggests that LAMs originate from bone marrow-derived infiltrating macrophages [24]; however, recent studies have also provided evidence that under some conditions, KCs themselves, either emKCs or moKCs, may also take on some of the phenotypic characteristics of LAMs and become more capable of handling steatosis-related stress. These specialized KCs have been termed “LAM-like KCs” or LLKCs [57] due to their expression of KC-specific markers such as VSIG4 [58], CLEC4F [59], CD163 [33,60], and TIMD4 [33,60] as well as LAM markers such as TREM2 and GPNMB. The frequent presence of TIMD4 in these cells bears similarity to the expression pattern of emKCs and suggests that some of them may originate from this cell type [30,33]. Tran et al. found an altered transcriptomic profile of isolated emKCs (defined as CLEC2+, TIMD4+) from MASH mice compared with emKCs isolated from chow-fed mice [20]. Specifically, MASH emKCs increased their expression of genes related to lipid handling including *Cd36* and *Pparg*, but maintained expression of KC markers such as *Cd163* and *Vsig4*. Interestingly, an increase in liver injury and a decrease in hepatic triglyceride storage was observed when emKCs were ablated, allowing moKCs (CLEC2+, TIMD4-) to dominate the hepatic macrophage pool. This suggests that the adapted emKCs play a dual role in promoting lipid accumulation as well as preventing inflammation in the steatotic liver [20]. Blériot et al. also identified two separate emKC populations (KC1 and KC2; distinguished by their expression of CD206) in a MASH murine model [61]. Using single-cell RNA sequencing, they found KC2 to be enriched in lipid processing genes such as *Cd36* and various fatty acid binding protein-related genes. Importantly, they confirmed the ontological identity of KC2s as being embryonically derived resident macrophages by using *Ms4a3*-TdT lineage tracing [62] methods which readily identify monocyte-derived myeloid cells [61].

Whether emKC-derived macrophages are present in hCLSs, whether LLKCs derive from emKCs, and exactly what function the LLKCs of the CLS play are all open questions. Itoh et al. were the first to suggest the presence of emKCs in hCLSs by using CD169, a lectin-type receptor enriched in tissue-resident macrophages which was present in hCLSs [7]. However, it is now known that CD169 can also be present in monocyte-derived macrophages as well [63]. De Ponti et al. hypothesized that emKCs can transition toward an LLKC phenotype [24], though this finding has not been widely confirmed. Thus, the hCLS LLKCs may arise from two different lineages. The function of these specific hCLS cells are also uncertain. The loss of CD169+ cells by DT-mediated ablation led to a significant reduction in fibrosis and the absence of hCLSs, suggesting a profibrotic role in the liver [7]. In a study of human MASH, CD163+ hCLSs were present in patients with more severe forms of steatohepatitis. In mouse liver, *Cd163* is primarily expressed in emKCs but whether CD163 expression provides any lineage information in human hepatic macrophages is unclear [64].

## 4. Factors That Modulate hCLS Development

### 4.1. Lipids

The accumulation of lipid droplets in hepatocytes is the hallmark of hepatic steatosis [65]. During steady-state conditions, hepatocytes maintain a homeostatic environment by balancing the formation, storage and utilization of lipids [66]. Lipid degradation in hepatocytes plays an important role in homeostasis and occurs via a combination of lipolysis and lipophagy [67,68]. These highly controlled processes are dysregulated in states of excess dietary consumption and obesity, leading to lipid droplet accumulation and enlargement. As the lipid-laden liver transitions from steatosis to steatohepatitis, several changes occur. These include the accumulation of toxic lipid products, hepatocyte ballooning and death [69,70], release of DAMPs, and influx of inflammatory macrophages [71]. The exact tipping point at which a shift in the hepatic microenvironment leads to this more inflammatory steatohepatitis state is unclear. It is clear, however, that the onset of steatohepatitis is necessary for formation of hCLSs [6,72].

The formation of CLSs appears to partially result from effects of both cholesterol and free fatty acids. An elegant study by Sakuma et al. showed that cholesterol in massive lipid droplets drives hepatocyte death and subsequent hCLS formation [73]. Fatty acids, likely released from dying lipid-laden hepatocytes, also play a role in this process. CD36 expressed on KCs binds to free fatty acids leading to progression of liver injury in MASH models [74]. These CD36-expressing KCs have been shown to preferentially accumulate intracellular lipids [75]. CD36 is prominently expressed on the macrophages that make up adipose CLSs [76], and liver LAMs also express CD36 [6,77,78]. Using single-cell transcriptomic approaches, De Ponti and colleagues found that both LAMs and LLKCs express *Cd36* and expression is highest in LLKCs [24]. However, the presence of CD36-expressing macrophages has not been definitively shown in hCLSs.

Adipocytes are an important source of free fatty acids and hepatic lipid droplets are strongly affected by fatty acid availability from adipocytes. Adipocyte death and consequent free fatty acid release lead to macrophage accumulation in the liver. The inhibition of adipocyte death through BCL-2 overexpression was found to reduce both lipid accumulation and CLS formation within the liver [79]. Thus, lipid-derived signals, in the context of both hepatocyte and adipocyte injury, seem to be critical for both the recruitment of macrophages and their formation into hCLSs.

### 4.2. FXR

Activation of the Farsenoid X Receptor (FXR) in hepatocytes is a key driver of lipid handling in the liver [80] and administration of the FXR agonist obeticholic acid (OCA) was found to decrease the presence of hCLSs in models of steatotic liver disease induced by high-fat diet [81]. In this same study, it was also found that OCA shifted hepatic macrophages toward a more protective and anti-inflammatory phenotype [81]. In a large transcriptomic study, OCA-mediated FXR activation in murine models of MASH was shown to regulate multiple macrophage-specific genes (i.e., *Itgal*, *Axl*, *Vcam1*) [82]. These findings highlight the critical nature of FXR activation in both altering macrophage phenotype and the development of hCLS. FXR activation is further mediated by the intestinal microbiota [83]. Antimicrobial depletion of specific bacteria in a murine model of MASH was found to increase formation of hCLSs and fibrosis as well as alter bile acid handling genes such as *Nr1h4* (FXR) and *Gpbar1* (TGR5) [84,85]. Altogether, altered bile acid composition secondary to diet and microbiome changes may play a role in hCLS formation in an FXR-mediated manner, though more investigation is necessary to determine the specific signals that regulate this process.

### 4.3. Inflammasome and NLRP3 Signaling

The inflammasome is a cytosolic multimer that assembles in response to the cumulative effects of multiple MyD88-dependent and -independent extrinsic inflammatory signals [86] and its activation generally requires a PAMP-type signal such as LPS in addition to an environmental signal such as ion fluxes or crystal formation. Following its formation, the inflammasome activates downstream pathways leading to the release of critical cytokines such as Interleukin-1β and Interleukin-18, both of which contribute to immune cell recruitment and shift macrophages toward a more inflammatory phenotype [87,88]. The NLRP3 inflammasome has been shown to be a key mediator for the formation of hCLSs. Ioannou and colleagues found that cholesterol crystallization in ballooned, lipid-laden hepatocytes occurred specifically in sites of hCLSs [72]. From these studies it was suggested that cholesterol crystal-induced inflammasome activation led to NLRP3 signaling which promoted formation of hCLSs [89]. Interestingly, other findings showed that when NLRP3 was globally knocked out to prevent fibrosis and inflammation, there was an absence of F4/80+ hCLSs compared to mice with intact NLRP3 [90]. It is important to mention that this latter study was performed using the MCD diet which leads to steatotic hepatic injury without significant weight gain. Thus, it is possible that NLRP3 signaling is a more critical driver of hCLS formation in the MCD diet condition than in the situation of steatohepatitis induced by high-fat diet alone [91]. Another caveat is that mice with a myeloid-specific deletion of NLRP3 had reduced hepatic fibrosis when fed a MASH diet, but hCLSs were still observed [92]. This suggests that myeloid inflammasome signaling is not strictly necessary for hCLS formation and the presence of the inflammasome in other sites, like hepatocytes, may play a role as well.

## 5. Role of hCLS in Steatosis Generation

While lipid accumulation is a critical factor for the development of hCLSs, hCLSs may also functionally contribute to lipid accumulation in SLD. Itoh and colleagues found that the number of hCLSs correlated with the degree of hepatic steatosis in human MASH, specifically in those that demonstrated F2 fibrosis [6]. Additionally, macrophages chronically exposed to a HFD may produce signals that promote hepatocyte lipid accumulation, as one study revealed that supernatant from ex vivo CD11b macrophages from mice fed a high-fat diet led to accumulation of steatosis in primary hepatocytes isolated from mice fed a normal chow diet [93]. However, it is unclear whether macrophages specifically from sites of hCLSs were responsible for this lipid-accumulating effect. Blériot et al. found that CD36, a protein that is co-expressed in LAMs along with TREM2 and GPNMB [49], is expressed in a subpopulation of KCs in a murine model of high-fat diet-induced fatty liver [61]. Weight gain, steatosis, and metabolic derangement were completely ameliorated in this model following depletion of CD36-expressing KCs using a DT-mediated ablation approach. Furthermore, genetic silencing of *Cd36* in KCs protected mice from metabolic dysfunction. Though limited, these findings may suggest that hCLSs may specifically contribute to steatosis development, but more research is necessary to determine the specific signals generated from hCLS sites and which specific macrophages (LAMs or LLKCs) may specifically drive this process.

## 6. Role of hCLS in Inflammation

The pro-inflammatory state found within steatohepatitis is driven largely by a pathogenic macrophage phenotype in which polarization by various signals such as lipopolysaccharide (LPS) shapes macrophages to secrete pro-inflammatory signals such as cytokines. Additionally, persistent tissue inflammation, such as that seen in progressive SLD states, has been shown to generate a pro-inflammatory memory state in which IMs and KCs are primed to secrete inflammatory mediators with a diminished triggering signal [94]. The macrophages within the hCLS appear to play a role in modulating these inflammatory responses.

### 6.1. Evidence for Pro-Inflammatory Function of CLS

Findings from a study by Makiuchi et al. further reinforce the idea that hCLSs exhibit a pro-inflammatory phenotype [95]. In a murine model of non-obese MASH fed a high-fat/cholesterol/cholate diet, Makiuchi and colleagues found that increased chronicity of diet feeding led to increased TNFα expression which paralleled an increase in CD11c-expressing cells that were specifically identified at sites of hCLS accumulation. Furthermore, Ioannou and colleagues found that TNFα expression was colocalized to CD68-expressing macrophages which were localized to hCLSs [72]. Finally, Zhou et al. found that hCLSs may in fact promote inflammation via *Ms4a7* [96]. Using RNA in situ hybridization, they found *Ms4a7* RNA localized to TREM2+ hCLSs in HFD-fed mice and subsequent knockout of *Ms4a7* led to a reduction in inflammation as well as a total reduction in TREM2 and GPNMB in whole-liver protein analysis [96].

### 6.2. Evidence for Anti-Inflammatory Function of CLS

Conversely, some investigators have shown that macrophages specific to the hCLS may in fact exhibit an anti-inflammatory phenotype. Substantial evidence has shown that the protein TREM2, which is expressed in both LAMs and LLKCs [49], plays a critical role in attenuating inflammation in multiple diseases, including cardiovascular, neurological, and liver diseases [97–101]. Dong et al. found that preventing cleavage of TREM2 via lipid nanoparticle injection prevented inflammation in a murine MASH model by enhancing macrophage capabilities for efferocytosis [102]. Fredrickson et al. found an association between expansion of the TREM2+ LAM population and reduced inflammation in the liver [103]. Using single-cell transcriptomics, they found that in HFD-fed mice, vertical sleeve gastrectomy led to an expansion of TREM2+ LAMs. These LAMs had a reduction in pro-inflammatory genes including *Nfkb1* and *Il1a* [103]. Ganguly et al. found that the loss of TREM2 led to increased activated Caspase 1, IL1α, IL22, and MCP1 in a model of MASH [50]. Altogether, these data reinforce the notion that hCLSs are complex and dynamic sites and that whether they are pro- or anti-inflammatory is likely heavily dictated by the chronicity of injury in SLD as well as other factors.

## 7. Functional Role of hCLS in Fibrosis

Though hCLSs were originally described as being associated with collagen deposition and stellate cell activation [6], their role in fibrosis is not entirely clear as subsequent studies have shown hCLSs to be antifibrotic and associated with fibrosis resolution. The profibrotic effect of macrophages in the liver results primarily from the ability of macrophages to generate signals that promote stellate cell activation and collagen synthesis. Specific signal transduction from HSCs back to macrophages has also been described [104–106] and thus macrophages and HSCs engage in back-and-forth crosstalk that determines the phenotype of both cell types. hCLSs appear to be particularly important sites at which macrophage–HSC crosstalk occurs.

Chan and colleagues found that F4/80+ hCLSs were in close contact with HSCs that line the outside of the hCLS [107]. These hCLSs colocalized with both type 1 collagen and alpha smooth muscle actin, suggesting that HSC activation was particularly strong around the hCLS [107]. The metabolic state of HSCs has also been found to play a key role in dictating liver fibrosis by communicating with macrophages in the hCLS. Habibi and colleagues found that the mitochondrial pyruvate carrier (MPC) plays a key role in this cell–cell signaling, as HSC-specific knockdown of MPC protected mice fed a choline-deficient diet from hepatic fibrosis [108]. Using bulk-RNA sequencing, they identified a shift in the macrophage phenotype during HSC-specific mitochondrial pyruvate carrier knockout with an observed decrease in LAMs/c-LAMs and reduction in SPP1 expression in transitioning monocytes, highlighting the bidirectional relationship between HSCs and hepatic macrophages specifically associated with hCLSs [108]. Nonetheless, it is important to note that fibrosis generation in SLD is not limited to sites of hCLSs and it is likely that profibrogenic signals from hCLSs such as TGFβ1 are secreted into other areas of the hepatic parenchyma as well [106].

### 7.1. Profibrotic Functions of hCLS

Several studies have shown a correlation between the presence of hCLSs and fibrosis generation. By depleting CD11c-expressing macrophages, Itoh et al. found a reduction in the total number of hCLSs which reappeared 4 days later [7]. This recurrence phase of the hCLSs led to an increase in whole-liver mRNA expression of profibrogenic signals such as Tgfb1, suggesting a correlative relationship between hCLSs and induction of collagen production. Another study showed that CD11c-expressing macrophages in hCLSs colocalized with collagen fibers [9]. Transcriptomic analysis in this study found that CD11c+ macrophages highly expressed genes related to liver fibrosis when compared to CD11c-macrophages, further supporting a profibrotic role of CD11c-expressing hCLS [9]. Itoh et al. also showed that iron stores regulate the ability of CD11c+ macrophages localized to hCLSs to take on a profibrogenic phenotype [8]. Altogether, these studies suggest that CD11c+ hCLSs may display a “wound healing” macrophage phenotype that is profibrotic.

### 7.2. Role of CLS in Fibrosis Resolution

Other evidence strongly suggests a role of hCLSs in fibrosis resolution. In Ccr2 knockout mice, Daemen et al. showed that the reduction in hCLSs was associated with an increase in fibrosis [11]. These investigators showed that Cathepsin K, a collagen protease, was specifically present in LAMs of the hCLS. It localized only to hCLSs in mice with intact CCR2, suggesting that at least some hCLS macrophages possess a fibrolytic phenotype [107]. Furthermore, TREM2-expressing macrophages, which are also present mainly in hCLSs, have been shown to demonstrate fibrosis-degrading properties as knockdown of Trem2 increased collagen deposition in the liver [50]. The loss of TREM2 in this study did not prevent the formation of hCLSs as GPNMB-expressing hCLSs were still present. In fact, the presence of CD11b+ macrophages increased in hCLSs, further highlighting the heterogenous nature of hCLSs [50]. These authors found that the hCLS cells expressed TREM2 and had a collagen degradation phenotype, and the absence of TREM2 restricted both the formation of hCLSs and collagen degradation.

Specialized hepatocytes surrounding the hCLS have also been found to promote a fibrosis resolution phenotype in macrophages. Using spatial transcriptomic studies in a murine model of alcohol-associated liver disease, Tikhanovich and colleagues found that hepatocyte-derived serum amyloid A (SAA) is a critical mediator of fibrosis degradation during alcohol resolution. In both murine and human models of alcohol liver disease, SAA-producing hepatocytes surrounded hCLSs and induced expression of proteases such as MMP12 and Cathepsin D in the CLS macrophages that were adjacent to SAA-expressing hepatocytes [109]. In another study, these authors found that KDM5B, a demethylase responsible for transcriptional repression, prevented fibrosis regression after alcohol cessation in part by regulating the hepatocellular production of oxysterols [110]. Unlike wild-type mice, hepatocyte-specific *Kdm5b* knockout mice showed robust fibrosis resolution after alcohol cessation. Using single-cell ATACseq, chromatin immunoprecipitation assays, and hepatocyte–macrophage co-culture studies, Tihhanovich and colleagues determined that *Kdm5b* knockout increased hepatocellular oxysterol production which activated LXR-mediated fibrosis degradation genes in macrophages including MMP12 and MMP9 [110]. These studies highlight a critical hepatocyte–macrophage relationship in which hepatocyte-derived products induce a fibrosis resolution phenotype macrophage which appears to be localized to sites of hCLSs.

## 8. Conclusions

**Figure 1.**
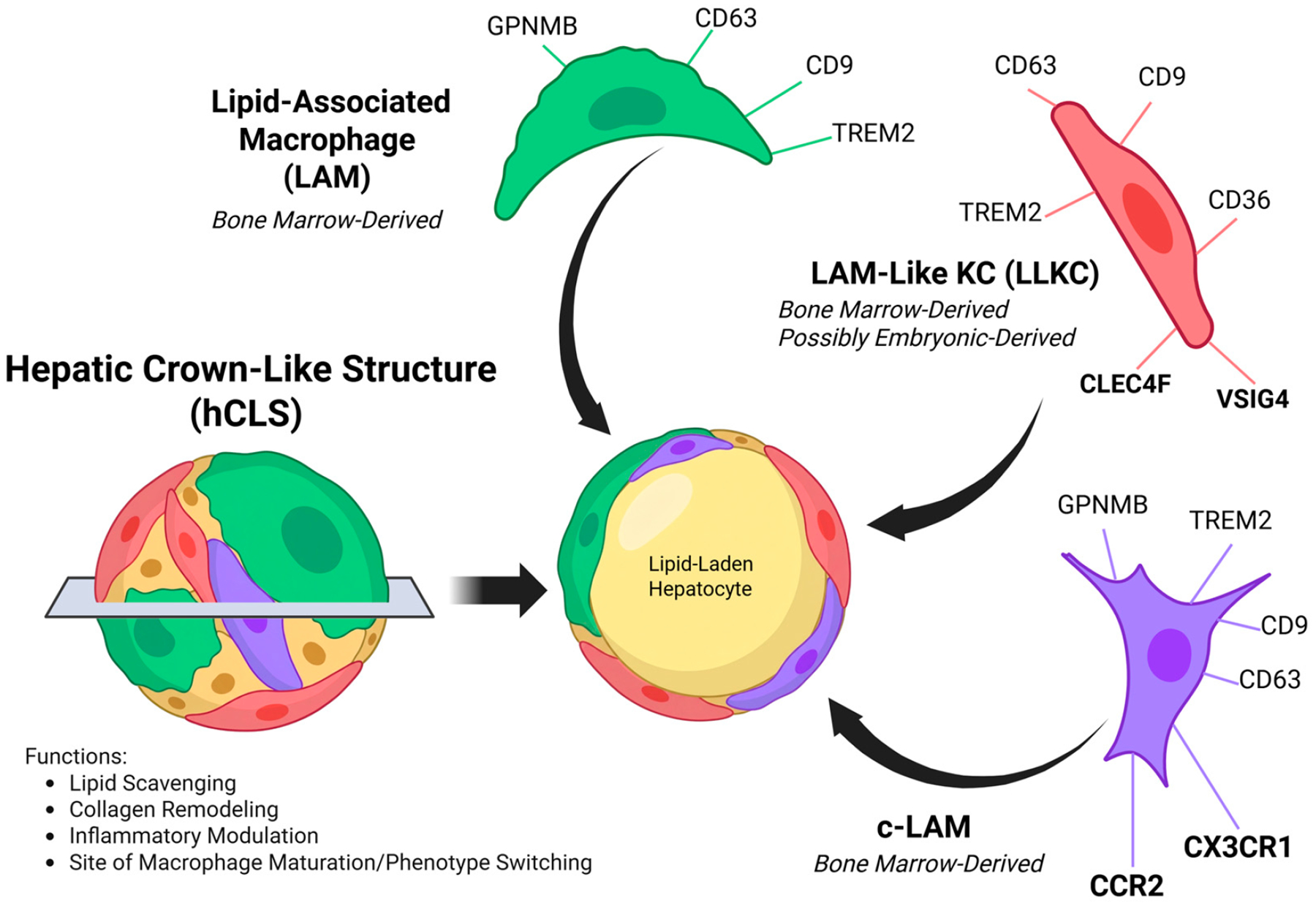
Schematic representation of the hepatic crown-like structure. At least 3 major cell types are present: classical LAMs, more inflammatory c-LAMs, and LAM-like Kupffer cells. Cell-type proteins, ontological origin, and functions are described. Created in BioRender. Pulido Ruiz, I.A. (2026) https://BioRender.com/u9mu3mz, accessed on 26 February 2026.

Our current concept of hCLSs is illustrated in Figure 1. hCLSs are complex, heterogeneous structures. They consist of large lipid droplets that occupy the cytosol of a dying or dead hepatocyte. These are surrounded by usually one layer of macrophages that covers the surface of the spherical structure and, when viewed in cross section, appears to be a ring of macrophages surrounding a central lipid droplet. They are composed of multiple different macrophage types that can perform multiple different functions. These include processing and degrading lipids, modulating inflammation and coordinating macrophage-dependent cell–cell interactions for both profibrotic and fibrosis-degrading purposes. The phenotypes of these specialized macrophages appear to be determined by multiple signals derived from hepatocytes, hepatic stellate cells, and the lipid itself. Finally, in addition to their functional importance, hCLSs appear to be sites at which liver macrophages are exposed to the signals that guide their phenotypic progression from monocytes to LAMs, LAM-like KCs and other macrophage types as well.

Many questions remain unanswered including whether bone marrow-derived infiltrating macrophages that compose the hCLS eventually leave these sites to become sinusoidal moKCs, whether emKCs move from the sinusoid to take on an LAM-like phenotype in the hCLS, and the precise role that each of these cell types plays in steatotic liver disease progression and resolution. Further studies that examine the formation and functions of the cells of the hepatic crown-like structure may provide new insights into ways to mitigate disease severity and enhance recovery for the spectrum of steatotic liver diseases, including alcohol-related liver disease. Further elucidating and possibly leveraging specific macrophage signals procured from hCLS sites which dampen HSC activation may lead to new targetable pathways and therapies.

## Figures and Tables

**Table 1.** Murine protein markers that characterize different liver macrophage subtypes in steatotic liver diseases.

IMs	moKCs	emKCs
CD68	CD68	CD68
F4/80	F4/80	F4/80
IBA1	IBA1	IBA1
CCR2	CCR2	VSIG4
CX3CR1	CX3CR1	CLEC4F
LY6C2	VSIG4	CD163
MINCLE/CLEC4E	CLEC4F	TIM4
	CLEC2	CLEC2
	CD169	CD169

IMs—Infiltrating Macrophages; moKCs—Monocyte-derived Kupffer cells; emKCs—Embryonic-derived Kupffer cells.

**Table 2.** Murine protein markers present in the macrophage subtypes that compose the hCLS.

LAMs	c-LAMs	LLKCs
CD11c	CD11c	CD11c
GPNMB	GPNMB	GPNMB
CD63	CD63	CD63
CD9	CD9	CD9
TREM2	TREM2	TREM2
CD36	CCR2	CD36
	CX3CR1	CLEC4F
		VSIG4
		CD169

LAMs—Lipid-Associated Macrophages; c-LAMs—Conventional Lipid-Associated Macrophages; LLKCs—LAM-Like Kupffer Cells.

## Data Availability

No new data were created or analyzed in this study. Data sharing is not applicable to this article.

## Abbreviations

Axl: AXL receptor tyrosine kinase
Vsig4: V-set immunoglobulin domain-containing 4
Ccr2: Chemokine (C-C motif) receptor 2
Cd163: Cluster of differentiation 163
Cd36: Cluster of differentiation 36
Gpbar1: G protein-coupled bile acid receptor 1
Gpnmb: Glycoprotein non-metastatic melanoma protein B
Il1a: Interluekin-1 alpha
Itgal: Integrin subunit alpha L
Kdm5b: Lysine demethylase 5B
Ms4a3: Membrane-spanning 4-domains subfamily A member 3
Ms4a7: Membrane-spanning 4-domains subfamily A member 7
Nfkb1: Nuclear factor NF-kappa-B p105 subunit
Nr1h4: Nuclear receptor subfamily 1 group H member 4
Ppar-gamma: Peroxisome proliferator-activated receptor gamma
Tgfb1: Transforming growth factor beta 1
Timd4: T-cell membrane protein 4
Trem2: Triggering receptor expressed on myeloid cells 2
Vcam1: Vascular cell adhesion molecule 1
